# Effect of feedback-enhanced reflective journaling on self-regulation and perceived performance among Taekwondo athletes

**DOI:** 10.1186/s40359-026-04575-6

**Published:** 2026-04-28

**Authors:** Ryu Seok, Sung-Un Park, Jeong-Hyeon Kwak

**Affiliations:** 1 Department of Taekwondo, Kyung Hee University, 17104, Yongin-Si, Republic of Korea; 2 Department of Sports Science, Hwasung Medi-Science University, Hwaseong-Si, 18274 Republic of Korea; 3 Graduate School of Education, Gachon University, Seongnam-Si, 13120 Republic of Korea

**Keywords:** Feedback-enhanced reflective journaling, Athlete self-regulation, Perceived performance, Sports psychology

## Abstract

**Background:**

Reflective journaling has been used as a strategy to help athletes examine their training experiences and support reflective learning. However, whether reflective journaling alone can produce meaningful changes in sport-related psychological outcomes remains unclear. Thus, this study explored whether feedback-enhanced reflective journaling (i.e., reflective journaling with structured feedback) would lead to greater improvements in self-regulation and perceived performance, conceptualized as athletes’ subjective self-evaluative beliefs, than reflective journaling alone.

**Methods:**

A total of 52 Taekwondo (Poomsae) athletes participated in the study and were randomly assigned to an experimental (*n* = 26) or control group (*n* = 26). For 16 weeks, both groups completed reflective journaling according to the same guidelines. The experimental group received structured feedback on their journal entries, whereas the control group did not. The journaling procedures, based on Gibbs’ six-stage model, focused on training reflection and personal insights. Pre- and post-tests assessed self-regulation and perceived performance. Statistical analyses—including independent-samples *t-*tests and two-way repeated-measures analysis of variance (ANOVA)—were conducted using the Statistical Package for the Social Sciences 23.0 (*α* = .05).

**Results:**

The experimental group showed significantly higher self-regulation (M = 4.214) and perceived performance scores (M = 3.900) compared with the control group (M = 3.764 and M = 2.800, respectively), with strong statistical significance (*p* < .01 and *p* < .001, respectively). The two-way ANOVA confirmed significant group × time interaction effects for both self-regulation (F = 12.013, *p* < .01) and perceived performance (F = 45.125, *p* < .001), suggesting that the addition of structured feedback to reflective journaling was associated with greater improvements in these outcomes.

**Conclusions:**

Feedback-enhanced reflective journaling was associated with greater improvements in self-regulation and perceived performance than reflective journaling alone among Taekwondo athletes. These findings highlight the potential value of adding structured feedback to reflective journaling in training programs to support athletes’ psychological development and more positive performance-related self-evaluations. The study offers practical implications for the development of sport-based psychological interventions targeting self-regulation and perceived performance.

**Trial registration:**

CRIS, KCT0011673, Retrospectively Registered March 3, 2026 [https://cris.nih.go.kr/cris/search/detailSearch.do?seq=32687&search_page=L&search_lang=&class_yn=].

## Background

Reflection is a powerful tool that can transform experiences into valuable insights, driving intellectual and emotional growth [1]. Reflection empowers individuals to take control of their learning journey, thereby fostering a deeper understanding of their actions and enhancing expertise in their field [2, 3]. In education, reflection is not just important, it is essential; reflection enables learners to critically assess their experiences, extract meaningful lessons, and apply these insights to improve future performance [4, 5]. This process is also relevant in sport, where reflection may help athletes internalize training experiences and better understand the thoughts, emotions, and behaviors associated with performance [6, 7].

Reflection is central to developing competence through deliberate practice and feedback. Research shows that top athletes consistently engage in high levels of reflective activity [6], suggesting that reflection is a critical element of athlete development that supports navigating challenges, making informed decisions, and demonstrating continuous improvement [8, 9]. Reflective journaling has been widely used to support reflective activity [3, 10]. However, whether reflective journaling alone is sufficient to produce meaningful changes in sport-related psychological outcomes, or whether any added benefit is primarily attributable to the provision of structured feedback, remains unclear.

Previous research suggests that reflective journaling may be associated with stronger reflective abilities [10–12]; expertise and growth [13–16]; and aspects of self-regulated learning [17, 18], metacognition [2], and learning strategies [2, 17]. Moreover, by prompting emotional reflection, reflective journaling advances reflective thinking, reasoning, self-awareness, interpersonal understanding, and the broader cognitive and emotional dimensions of learning [19, 20]. Structured feedback may promote reflection by helping individuals develop a clearer understanding of their experiences and engage in more focused and meaningful reflective processes [21–23]. Taken together, these findings suggest that reflective journaling may provide a useful reflective structure, but that structured feedback may be the more active ingredient in helping athletes interpret their experiences in ways that support learning and development.

From a self-regulation perspective, this possibility can be explained more clearly. Self-regulation refers to the self-generated cyclical control of thoughts, emotions, and behaviors directed toward valued goals [24, 25]. Individuals determine desired end states; monitor their progress; and adjust their cognition, emotions, and behaviors accordingly [24, 25]. In sport contexts, self-regulation is essential for adaptation, well-being, and performance-related functioning, particularly among elite athletes [9, 26, 27]. Maintaining self-regulation requires core components such as self-awareness [24], which enables athletes to understand their internal states and regulate the psychological and physiological conditions necessary for effective performance and recovery [28–30].

Reflective journaling may provide an initial platform for this process by externalizing athletes’ training experiences, thoughts, and emotions, whereas structured feedback may help transform those reflections into more focused self-awareness and enable the metacognitive monitoring of performance-relevant states [2, 3, 28, 31–35]. Expressive and reflective writing has been shown to facilitate cognitive and emotional regulation [34–36]. When reflection is supplemented with structured feedback, it may act as external scaffolding, helping athletes interpret discrepancies between intended goals and current states, regulate emotional responses, and formulate more concrete plans for subsequent training [21–23, 37, 38]. Thus, the added structured feedback may be what enables reflective journaling to function not merely as a record of experience but as a cyclical self-regulatory process involving monitoring, evaluation, and behavioral adjustment [24, 25]. Through repeated engagement in this feedback-supported cycle, athletes may strengthen self-regulation and develop more positive self-evaluative beliefs regarding their competence and readiness [29, 39, 40].

In the present study, these self-evaluative beliefs were conceptualized as perceived performance, defined as athletes’ subjective self-evaluative beliefs about their current competence, readiness, and mastery within the sport rather than an objective indicator of athletic performance [40, 41]. These perceived performance beliefs are important in sport because athletes’ subjective evaluations of their competence, readiness, and performance effectiveness may influence confidence, effort regulation, and subsequent performance-related behaviors [26, 29, 40]. In applied sport settings, even in the absence of immediate changes in objective competition outcomes, such self-evaluative judgments may serve as proximal indicators of how athletes perceive and interpret their current performance states [40, 41]. Previous research has examined perceived performance, performance satisfaction, and psychological perceptions of performance readiness and effectiveness as variables reflecting the self-evaluative aspects of performance [29, 41, 42]. The findings suggest that self-evaluations of performance-related states may constitute a meaningful psychological outcome in sport.

Accordingly, the present study did not assess objective athletic performance; rather, it examined whether the intervention was associated with changes in athletes’ subjective self-evaluative beliefs about their competence, readiness, and mastery within the sport. Although related constructs such as competitiveness have been discussed in the sports psychology literature [43], this study focused specifically on perceived performance. Any incremental effects of adding structured feedback to reflective journaling were expected to be reflected more readily in athletes’ self-regulatory and evaluative processes than in direct changes in objective performance.

Sports science has long been concerned with identifying psychological factors that shape athletes’ functioning and development [9, 26, 27]. While performance remains important, the goals of sports science extend beyond performance to encompass optimal performance, development, and enjoyment [9, 26, 27, 44, 45]. Psychological interventions for elite athletes have been shown to promote recovery from injuries [46] and enhance performance-related outcomes [47], while simultaneously contributing to broader outcomes related to personal growth and well-being. The findings suggest that athletes’ psychological functioning can be improved through targeted interventions [46, 47], thereby encouraging further research on psychological processes related to sport performance and development [47–50]. Furthermore, previous studies have highlighted the importance of emotion regulation, mental health, and social connectedness in shaping athlete outcomes [34, 51–54], suggesting that emotional reflection and regulation may represent one pathway through which feedback-enhanced reflective journaling exerts its effects.

Based on this theoretical rationale, this study examined whether the addition of structured feedback to reflective journaling would lead to greater improvements in self-regulation and perceived performance than reflective journaling alone among Taekwondo athletes. This study aimed to clarify the added value of structured feedback within reflective journaling and to contribute to the development of psychologically informed interventions in sport. The following hypotheses were tested.

- H1. Compared with reflective journaling alone, 16 weeks of reflective journaling with structured feedback will result in greater increases in self-regulation and perceived performance in the experimental group.
- H2. A significant time (pre–post) × group (feedback-enhanced reflective journaling vs. reflective journaling alone) interaction effect for both self-regulation (H2-a) and perceived performance (H2-b) will be observed.

**Fig. 1:**
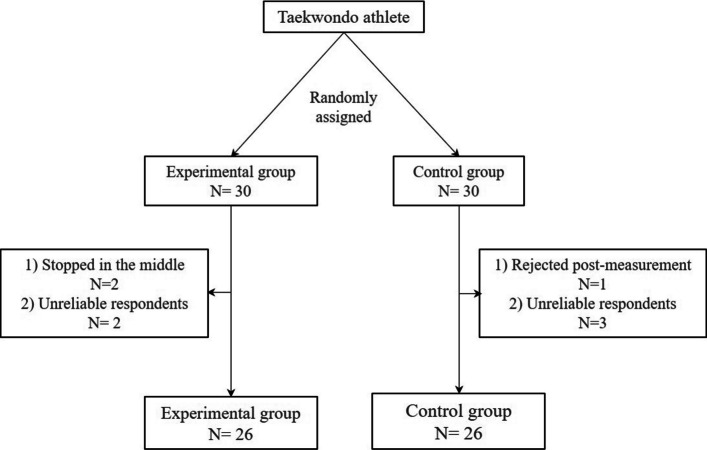
Research model

Figure 1 presents the research model.

## Methods

### Procedures

We employed a pre-test–post-test experimental design with random participant assignment. The pre-test was conducted on July 25, 2022, and the post-test was conducted 16 weeks later, on November 18, 2022. The independent variable was the provision of structured feedback during the reflective journaling process. Participants were randomly assigned to either the experimental or control group.

Although Taekwondo competitions are conducted individually, training generally takes place in a team setting. Accordingly, the control and experimental groups belonged to the same team, received instruction from the same coach, and participated in identical training sessions throughout the study period. Therefore, the frequency, duration, and types of physical activities remained the same for both groups. The primary between-group difference was that the experimental group received researcher-provided feedback on their reflective journals, whereas the control group completed journaling without such feedback. This design was intended to minimize the influence of extraneous variables and to make the provision of structured feedback the primary differentiating factor between the two groups. To ensure systematic reflection, participants received orientation on journal writing procedures prior to the intervention.

### Intervention procedure

The intervention procedure was designed to examine whether the addition of individualized researcher feedback within reflective journaling would be associated with greater improvements in self-regulation and perceived performance than reflective journaling alone. All participants were instructed to complete a reflective training journal according to the provided guidelines. Participants were instructed to complete journal entries at least three times per week throughout the 16-week intervention period. Adherence was monitored using journal submission records, and participants were expected to complete a total of 48 entries. Among the final analytic sample, all 52 participants completed all 48 expected entries. In the experimental group, structured feedback was provided for 100% of submitted journal entries. Although entries were written in free narrative form, the journaling process was guided by a structured reflection framework with predefined sections and question prompts.

The reflective journal consisted of three main sections: (1) background questions (e.g., condition, training intensity, areas of pain, notable issues); (2) reflective activities (e.g., training theme and goals, personal goals for the day, training content and methods, reflection on training content, application and contribution of the training, personal insights); and (3) overall reflection (e.g., a message to oneself for the day, training satisfaction scale). In addition to the orientation, detailed guidelines for completing the journal were provided. The reflective journal used in this study was adapted and refined from Seok’s [55] version for Taekwondo (Poomsae) athletes, which is based on the six-stage reflective model proposed by Gibbs [56]. The structured framework entitled “Reflection framework: steps, key elements, and guiding questions” (see Table 1) supported this process by providing question prompts to facilitate reflection. Participants used the framework as a guide while writing their entries in an open narrative format. Although it was not mandatory to respond to every question, participants were encouraged to follow the guidelines and expand upon emerging thoughts to broaden the scope of their reflection. Details of the reflective journal are presented in Table 1.

**Table 1:** Reflection framework: steps, key elements, and guiding questions

Step		Content		Item
*Step 1*	Description	Detailing the experience	Training content and methods	▶ What happened? ▶ When and where did it happen? ▶ Who did you train with? ▶ What did you and others do? ▶ What was the result of the training? ▶ Why were you there? ▶ What were you hoping would happen?
*Step 2*	Feelings	Expressing feelings and thoughts about the experience	Reflection on the training content	▶ What did you feel in that situation? ▶ How did you feel before and after the situation? ▶ How do you think other people feel about that situation? ▶ What do other people think about the situation now? ▶ What were you thinking in that situation? ▶ What do you think about the situation now?
*Step 3*	Evaluation	Evaluating good and bad experiences		▶ What were the good and bad aspects of the experience? ▶ What went well? ▶ What did not work? ▶ What did you and others contribute to the situation?
*Step 4*	Analysis	Analyzing the cause and effect of the incident	Reflection on the application and contribution of the training content	▶ Why do you think things went well? ▶ Why do you think things did not go well? ▶ What meaning can this situation have? ▶ What knowledge is helpful to me or to others to understand the situation?
*Step 5*	Conclusion	Drawing lessons from experience		▶ What have you learned from this situation? ▶ Could this have been a more positive situation for all involved? ▶ What skills do you need to develop to better handle situations like this? ▶ What else could you have done?
*Step 6*	Action plan	Developing an action plan for the future	Thoughts and future plans through reflection	▶ What would you do if you had to do the same thing again? ▶ How would you develop the essential skills you need? ▶ How can you make sure you do things differently next time?

The control group received only the journaling guidelines. Participants in the experimental group submitted each journal entry to the researchers on the day it was written and received individualized written feedback on each submitted entry throughout the intervention period. Written feedback was typically returned within 48 h of journal submission. To ensure consistency across participants, feedback followed a standardized structure consisting of three stages based on Hattie and Timperley’s [21] model: feed-up (clarifying goals and performance standards), feedback (reviewing self-evaluated performance-related experiences and identifying causes), and feedforward (planning strategies for improvement in subsequent sessions). These feedback elements were aligned with the reflection framework to support deeper reflection and encourage the development of self-regulation strategies.

All feedback was provided by the same researcher, and although the content was individualized to each participant’s reflections, the format and core components were standardized across participants. All feedback addressed common elements, including goal clarification, evaluation of current training experiences and performance-related challenges, and suggestions for subsequent improvement.

Both groups completed reflective journaling under the same guidelines. The only systematic difference between the conditions was the inclusion of researcher-provided structured feedback for the experimental group. Accordingly, the study design examined the added effect of structured feedback during the reflective journaling process rather than the effect of reflective journaling per se.

### Participants

We calculated the required sample size using G*Power software (version 3.1.7; Heinrich-Heine University, Düsseldorf, Germany) for a 2 × 2 repeated-measures analysis of variance (ANOVA). Assuming an effect size of 0.25, a significance level (α) of 0.05, and a statistical power (1-β) of 0.80, the minimum required sample size was estimated to be 34 participants. To ensure sufficient statistical power and to account for potential dropouts—particularly given the reflective journaling requirement and the objective of detecting between-group differences in self-regulation and perceived performance—we aimed to recruit 60 participants.

A total of 60 elite Taekwondo athletes aged 18 years or older were initially recruited. These athletes were all actively competing in university championships and national-level tournaments in Korea and registered with the Korea Taekwondo Association in 2022. Owing to personal reasons, eight participants dropped out during the study and were not included in the final analysis, leaving a final analytic sample of 52 participants. Participants were randomly assigned to either the control (*n* = 26) or experimental group (*n* = 26). The study was conducted without informing participants of their group assignments. The results of the chi-squared tests showed no significant differences between groups across variables such as sex, current academic year, and athletic experience (*p* > 0.05). These results are presented in Table 2.

**Table 2:** Results of testing the homogeneity in general characteristics between the two groups (*n* = 52)

Variable	Category	*n*	Percentage (%)	Group		χ2	*p*
				**Experimental**	**Control**		
Sex	Male	29	55.8	14	15	0.078	0.780
	Female	23	44.2	12	11		
Current academic year	1 st year	18	34.6	11	7	2.789	0.425
	2nd year	16	30.8	6	10		
	3rd year	8	15.4	3	5		
	4th year	10	19.2	6	4		
Athletic experience	5 years or less	13	25.0	6	7	1.210	0.546
	6–10 years	30	57.7	14	16		
	More than 10 years	9	17.3	6	3		
Total		52	100.0	26	26		

### Measures

Self-regulation was measured using seven items adapted from the Self-Regulation of Learning Self-Report Scale (SRL-SRS), originally developed and validated by Toering et al. [57] and later refined by Kim et al. [58]. Items were carefully selected based on their theoretical relevance to the study’s objectives and their applicability to the training environment of Taekwondo athletes. To ensure content validity and contextual appropriateness, a panel of three experts in sports psychology and Taekwondo performance reviewed the preliminary items. Based on their feedback, minor revisions were made to the wording and phrasing to improve clarity, cultural suitability, and domain specificity.

Perceived performance was measured using five items selected from the Task and Ego Orientation in Sport Questionnaire (TEOSQ), originally developed by Duda and Nicholls [59] and previously used in Korean sport contexts by Kim and Gill [41]. In the present study, item selection and wording were further refined based on the sub-evaluation framework proposed by Hersey and Blanchard [60]. Although the TEOSQ is traditionally used to assess goal orientations, the selected items (e.g., “I have top-level performance,” “I am a top-level athlete”) were operationalized to capture athletes’ subjective self-evaluative beliefs regarding their current competence, readiness, and mastery in the sport, rather than motivational orientation. The items were reviewed by domain experts to evaluate their relevance and content validity in the Taekwondo context. Revisions were made as needed to improve contextual appropriateness and clarity. This operationalization should be interpreted as a measure of self-evaluative perceived performance, not as a direct measure of competitiveness or objective performance.

Reliability was assessed using Cronbach’s α, with 0.80 considered the minimum threshold for acceptable internal consistency [61]. The SRL-SRS and TEOSQ demonstrated a Cronbach’s α of 0.891 and 0.902, respectively. Thus, both instruments indicated strong internal consistency.

Construct validity was examined using an exploratory factor analysis (EFA) of the pre-test data. Furthermore, varimax orthogonal rotation was applied to clarify factor loadings and assess data validity [62, 63]. The EFA criteria included a Kaiser–Meyer–Olkin (KMO) value above 0.50, a significant Bartlett’s test of sphericity, eigenvalues greater than 1.0, and factor loadings exceeding 0.40 [63–66]. The analysis demonstrated excellent sampling adequacy (KMO = 0.877; Bartlett’s test: χ^2^ = 819.501, *df* = 66, *p* < 0.001). A two-factor solution was extracted, with all factor loadings above 0.65, accounting for 66.89% of the cumulative variance, thereby surpassing the conventional 60% threshold for construct validity [61, 67]. These results suggest that the instruments showed preliminary evidence of validity and reliability for use with Taekwondo athletes. However, because the perceived performance items were adapted from a measure originally developed for goal orientation, this construct should be interpreted cautiously as a subjective self-evaluative measure of athletes’ perceived competence and performance-related readiness rather than a definitive indicator of objective athletic performance. The detailed results are presented in Table 3.

**Table 3:** Exploratory factor analysis of self-regulation and perceived performance

Items	Self-regulation	Perceived performance	
When I encounter problems related to exercise, I overcome them well	**0.842**		0.066
I cooperate well with my coach to improve my skills	**0.761**		0.343
The exercise method I use is appropriate	**0.754**		0.283
I usually follow my exercise plan well	**0.729**		0.349
Even when I am under excessive stress about exercise, I overcome it on my own	**0.674**		0.151
I tend to take the lead during practice	**0.667**		0.474
I maintain close relationships with my colleagues	**0.651**		0.354
I have top-level performance	0.174		**0.866**
I am a top-level athlete	0.207		**0.850**
My perceived performance is increasing	0.235		**0.782**
I always think I can play well	0.369		**0.753**
My perceived performance is satisfactory	0.378		**0.752**
Cronbach’s α	0.891		0.902
Eigenvalue	4.118		3.913
Explanatory variance (%)	34.320		32.605
Cumulative variance (%)	34.320		66.925
KMO = 0.877, *x*^*2*^ = 819.501, *df* = 66, *p* < 0.001			

### Statistical analysis

The collected data were analyzed using descriptive statistics, validity and reliability analyses, independent-samples *t-*tests, and two-way repeated ANOVA using the Statistical Package for the Social Sciences version 23.0 (IBM Corporation, Armonk, NY, USA). Before conducting all statistical analyses, the assumptions of normality and homogeneity of variance were tested using the Shapiro–Wilk and Levene’s tests, respectively. All statistical tests were conducted at a significance level of *p* = 0.05.

## Results

### Normality verification and descriptive statistics

As part of our analysis, we used the Shapiro–Wilk test to verify data normality. The results revealed that the *p*-values for the Shapiro–Wilk test were greater than 0.05 (*p* > 0.05), indicating that the data followed a normal distribution [68, 69]. The details of normality verification and descriptive statistics are presented in Table 4.

**Table 4:** Normality assessment for self-regulation and perceived performance

Factor			M	SD	*W*	*p-*value
Experimental	Pre	Self-regulation	3.330	0.633	0.926	0.064
		Perceived performance	2.415	0.660	0.960	0.391
	Post	Self-regulation	4.214	0.598	0.932	0.087
		Perceived performance	3.900	0.613	0.946	0.186
Control	Pre	Self-regulation	3.549	0.405	0.949	0.214
		Perceived performance	2.638	0.265	0.923	0.054
	Post	Self-regulation	3.764	0.221	0.934	0.095
		Perceived performance	2.800	0.358	0.946	0.184

*M* mean, *SD* Standard deviation, *W* Shapiro–Wilk test statistic

### Hypothesis testing

To verify H1, an independent *t*-test was conducted; the results are presented in Table 5.

**Table 5:** Independent* t*-test of post-intervention self-regulation and perceived performance

Group	Self-regulation		Perceived performance	
	**Experimental**	**Control**	**Experimental**	**Control**
*N*	26	26	26	26
M	4.214	3.764	3.900	2.800
SD	0.598	0.221	0.613	0.358
*t(p)*	3.602(0.001)		7.907(0.000)	
Cohen’s* d* (95% CI)	0.99 (0.42, 1.58)		2.19 (1.50, 2.89)	

The independent *t*-test was conducted using post-intervention data to examine group differences after the intervention. Cohen’s *d* represents the standardized mean difference, and 95% CI indicates the 95% confidence interval for Cohen’s *d*

The analysis revealed a statistically significant difference in self-regulation (*t* = 3.602, *p* < 0.01), with the experimental group (mean [M] = 4.214, standard deviation [SD] = 0.598) scoring higher than the control group (M = 3.764, SD = 0.221). The magnitude of this difference represented a large effect size (Cohen’s *d* = 0.99, 95% CI = 0.42, 1.58). Furthermore, a statistically significant difference was observed for perceived performance scores (*t* = 7.907, *p* < 0.001), indicating that the experimental group (M = 3.900, SD = 0.613) scored higher than the control group (M = 2.800, SD = 0.358). This result also showed a very large effect size (Cohen’s d = 2.19, 95% CI = 1.50, 2.89), which should be interpreted with caution given the self-reported and adapted nature of the perceived performance measure. These post-test differences support H1 and are consistent with the interpretation that the addition of structured feedback was associated with higher self-regulation and perceived performance scores.

Both groups completed reflective journaling; the critical experimental difference in this study was the provision of structured feedback. Therefore, the primary comparison was between reflective journaling with and without structured feedback. A two-way repeated-measures ANOVA was conducted to examine the interaction between group (experimental vs. control) and time (pre vs. post) in testing H2.

**Fig. 2:**
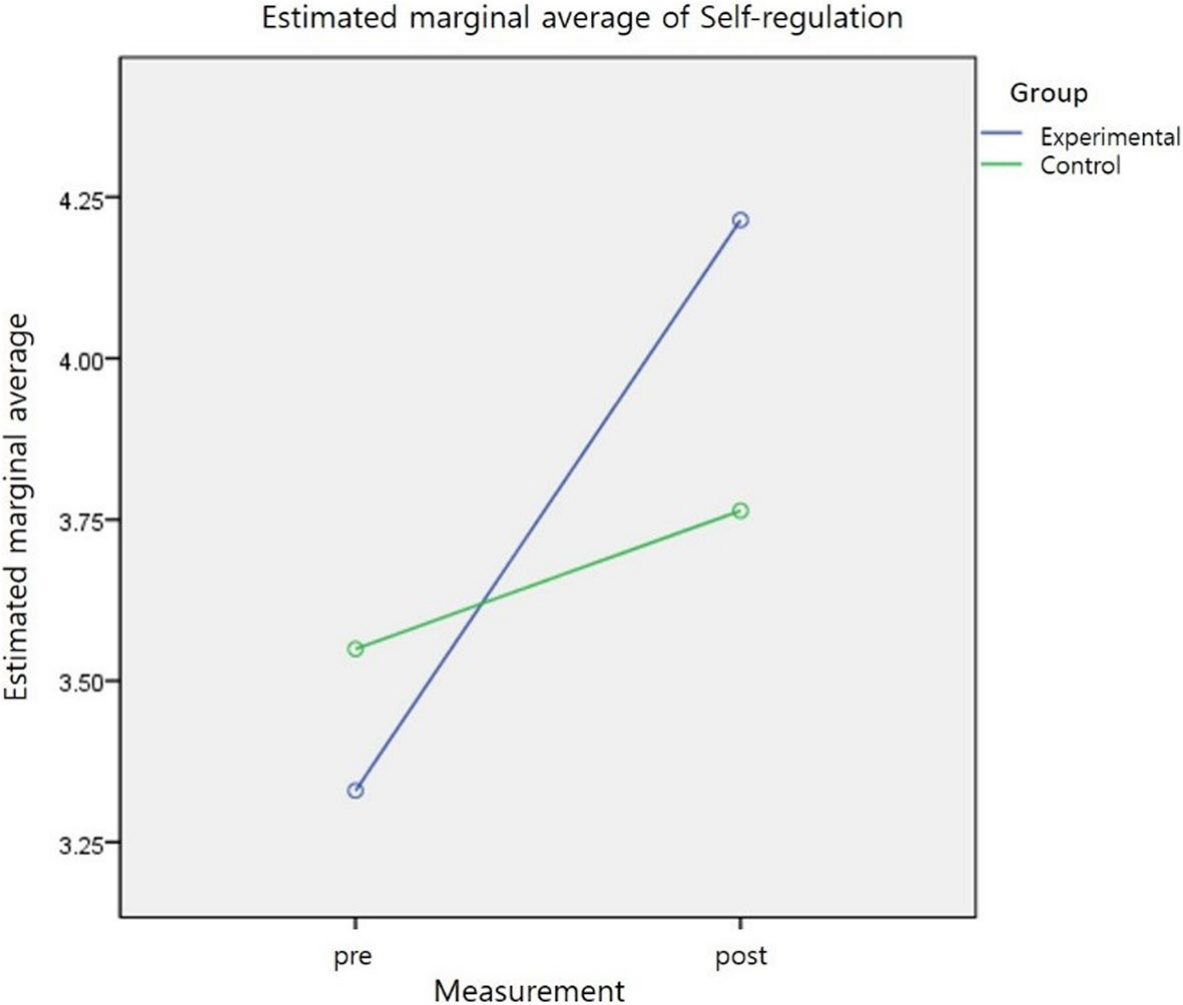
A two-way ANOVA analysis on self-regulation

The results are presented in Tables 6 and 7. The analysis of the interaction effect on self-regulation revealed a significant interaction between time (pre vs. post) and group (experimental vs. control), *F (1, 100)* = 12.013, *p* < 0.01, partial *η*^2^ = 0.107, as presented in Table 6 and Fig. 2. This interaction indicated that the experimental group, who received structured feedback, showed significantly greater improvement in self-regulation over time than the control group, supporting H2-a.

**Table 6:** Interaction effects of groups and measures (pre–post) on self-regulation

Independent variable	SS	*df*	MS	*F(p)*	*ηp*^*2*^
Adjusted model	11.116	3	3.705	15.241(0.000)	0.314
Intercept	1434.776	1	1434.776	5901.41(0.000)	0.983
Measure	7.849	1	7.849	32.285(0.000)	0.244
Group	0.346	1	0.346	1.424(0.236)	0.014
Measure * Group	2.921	1	2.921	12.013(0.001)	0.107
Error	24.31	100	0.243		
Total	1470.202	104			

*SS* sum of squares, *MS* mean square, *ηp*^2^ partial eta squared

**Table 7:** Interaction effects of groups and measures (pre–post) on perceived performance

Independent variable	SS	*df*	MS	*F(p)*	*ηp*^*2*^
Adjusted model	33.991	3	11.330	44.934(0.000)	0.574
Intercept	897.994	1	897.994	3561.293(0.000)	0.973
Measure	17.614	1	17.614	69.854(0.000)	0.411
Group	4.998	1	4.998	19.823(0.000)	0.165
Measure * Group	11.378	1	11.378	45.125(0.000)	0.311
Error	25.210	100	0.252		
Total	957.200	104			

*SS* sum of squares, *MS* mean square, *ηp*^2^ partial eta squared

**Fig. 3:**
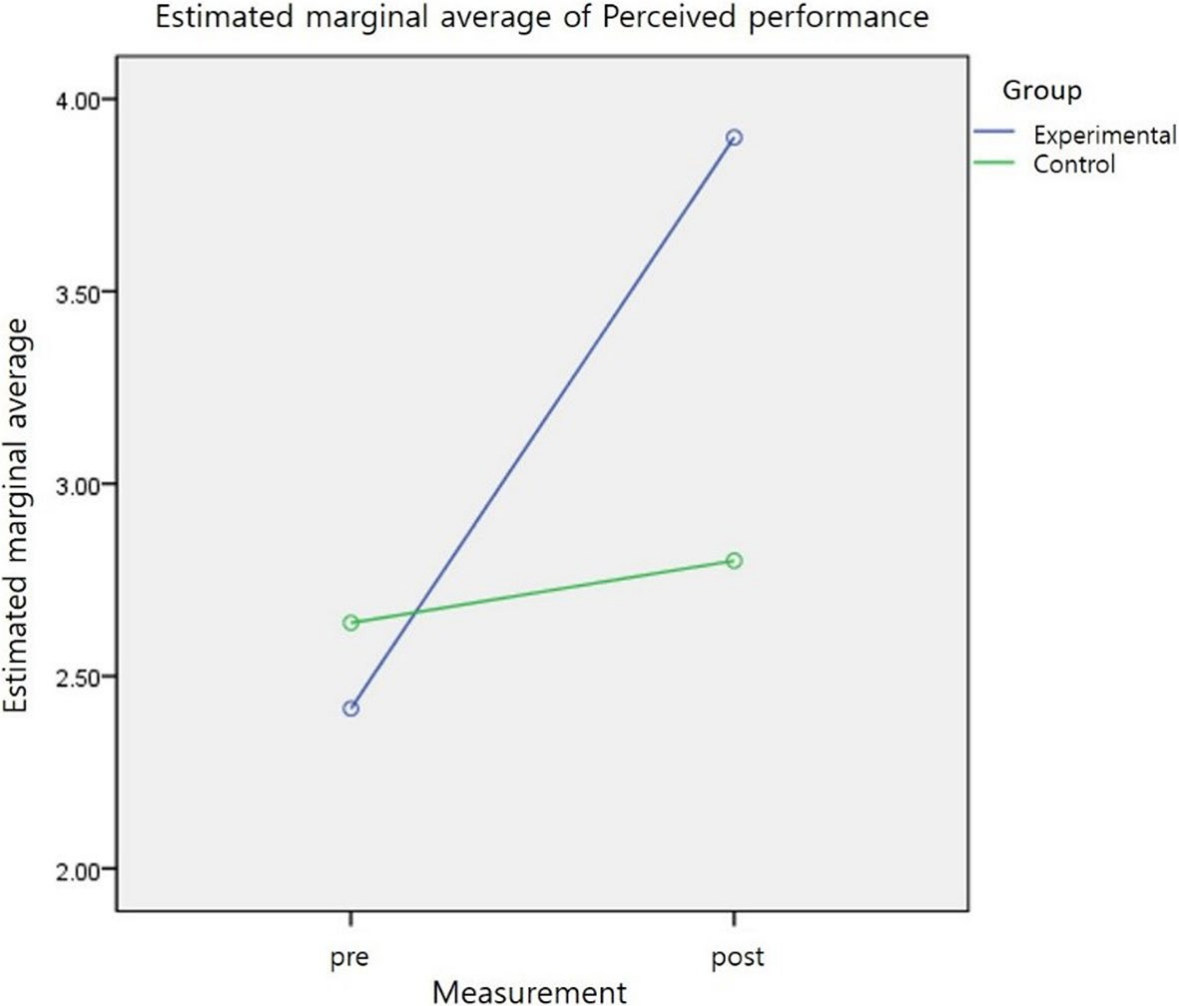
A two-way ANOVA analysis on perceived performance

The analysis of the interaction effect on perceived performance revealed a significant interaction between time (pre vs. post) and group (experimental vs. control), *F* (1, 100) = 45.125, *p* < 0.001, partial *η*^2^ = 0.311, as presented in Table 7 and Fig. 3. These results indicated that the experimental group, who received structured feedback, showed a significantly greater increase in perceived performance scores over time than the control group, supporting H2-b.

## Discussion

This study’s findings suggest that the addition of structured feedback within a reflective journaling process may contribute to higher self-regulation and perceived performance scores among Taekwondo athletes. These findings indicate that structured feedback may enhance the value of reflective journaling in sport settings. However, the findings’ generalizability to other sports and populations requires further investigation [70, 71]. While reflective journaling alone may provide a platform for self-discovery, the integration of expert feedback may help direct this process toward specific performance-related goals. Feedback-enhanced reflective journaling may facilitate the analysis and evaluation of personal training and competition experiences through writing combined with subsequent expert guidance. This collaborative process may help athletes identify their strengths and weaknesses more systematically and support the development of more targeted strategies [28, 71, 72]. Reflective journaling supported by feedback may also facilitate cognitive processing and emotional expression, which may help individuals reframe stressful events more positively and support self-regulation through improved management of thoughts and emotions [35]. Although the observed pattern is consistent with the possibility that structured feedback supported self-monitoring, self-evaluation, and behavioral adjustment, the present study did not directly measure metacognitive processes, reflective depth, or self-awareness. Therefore, this mechanism should be regarded as a plausible interpretation rather than an empirically tested pathway.

Self-reflection is a critical evaluative process that enhances the ability to detect problems and emulate strengths, which may contribute to more favorable performance-related perceptions and adaptive self-regulatory outcomes [72, 73]. When reinforced by external feedback, such structured reflective activities may serve as effective tools for enhancing perceived performance scores as well as sustaining effort and goal commitment by reducing subjective biases [40]. Therefore, the present findings are more appropriately interpreted as reflecting the added value of structured feedback within reflective journaling, rather than the independent effect of journaling alone. At the same time, the present design cannot determine whether the observed advantage was attributable specifically to the content of structured feedback, to additional interpersonal attention and accountability, or to expectancy-related effects associated with receiving individualized responses. Future studies should incorporate an attention-matched control condition to distinguish feedback-specific effects from non-specific intervention effects.

Self-regulation is a cyclical process involving self-monitoring, self-evaluation, and behavioral adjustment [24, 25, 74–77]. Feedback-enhanced reflective journaling may help athletes organize and strengthen these processes in a more systematic way [38]. Specifically, the feedback component may foster metacognitive strategies that enable athletes to assess their strengths and weaknesses against professional benchmarks, evaluate discrepancies between intended goals and current states, and adjust their learning and performance strategies accordingly [22, 23]. Thus, feedback-enhanced reflective journaling may serve as a practical self-regulation tool by facilitating deeper analysis of actions and experiences and by supporting the development of actionable plans to achieve goals [78, 79]. This process may help athletes make more informed decisions in competitive contexts. However, these self-regulatory and metacognitive processes were not directly assessed in the present study, and such interpretations should therefore remain tentative.

From an ecological dynamics perspective, self-regulation is critical for developing the adaptive behaviors (perceptual, cognitive, and motor-based) necessary to address performance challenges during competitions [80]. Furthermore, the interaction between cognitive load, external scaffolding through feedback, and self-regulation may significantly enhance self-regulatory abilities [81]. Feedback-enhanced reflective journaling may provide systematic support for self-regulation, helping athletes design effective action plans and develop adaptive behaviors for competition. Therefore, this “enhanced” element of journaling may represent an important component in supporting athletes’ interpretation of performance challenges and decision-making processes in competitive scenarios.

The addition of structured feedback within reflective journaling may also support higher perceived performance. By documenting and analyzing their experiences and receiving expert feedback on their psychological states, athletes may be better able to reflect on, define, and refine competition strategies [28]. This feedback-loop process may aid in managing emotions, reframing stressful experiences, and adjusting behaviors to achieve goals [35]. Guided reflective practices may also support self-regulation and help reduce the risk of injury and psychological burnout caused by overtraining, thereby supporting athletes’ performance-related adaptation [82, 83]. By gaining a greater understanding of and receiving external confirmation of their emotional and psychological states during training and competition, athletes may be better able to sustain self-monitoring and performance-related adaptations [82]. The adoption of feedback-enhanced reflective journaling may support psychological stability and perceived preparedness over time and may be consistent with the development of metacognitive strategies, although this was not directly tested in the present study. Accordingly, the observed improvement in perceived performance noted in this study is more likely to reflect more positive subjective self-evaluative beliefs about competence and readiness than direct gains in objective athletic performance [40, 84]. Accordingly, the magnitude of the observed effect on perceived performance should not be interpreted as evidence of equivalently large gains in objective athletic performance, but rather as a strong between-group difference on an adapted self-evaluative outcome within this sample.

Despite these contributions, this study had several limitations. First, the study focused exclusively on Taekwondo athletes, which may limit the generalizability of the findings to other sports or the general population. Second, the psychometric evaluation of the adapted measures should be considered preliminary; although the EFA produced acceptable loadings, the sample size (*N* = 52) relative to the 12 items stood at the lower boundary for stable factor extraction. Moreover, the adapted perceived performance measure should be interpreted as reflecting athletes’ subjective self-evaluative beliefs about their competence, readiness, and mastery, rather than as a direct indicator of objective performance or competitiveness. Accordingly, the observed effects on perceived performance may reflect changes in athletes’ performance perceptions rather than direct improvements in objective athletic performance. Furthermore, despite the pre–post design, no confirmatory factor analysis or formal test of measurement stability across time was conducted. Third, the experimental design introduced the potential for contamination or diffusion effects, as both groups belonged to the same team and trained under the same coach. The individualized feedback and additional attention provided to the experimental group may also have generated expectancy or accountability effects, which should be acknowledged when interpreting the results. As both groups completed reflective journaling and only the experimental group received structured feedback, the study design does not isolate the independent effect of reflective journaling itself; rather, the study evaluates the added value of structured feedback within a reflective journaling process. Fourth, the effectiveness of the intervention may vary depending on individual athletes’ levels of reflection and writing skills. Finally, while the study examined the effects over 16 weeks, it did not assess whether these effects were sustained over time. Future research should explore the long-term impact and potential for variation in intervention effects over extended periods.

## Conclusion

This study provides preliminary evidence regarding the added value of structured feedback within reflective journaling for self-regulation and perceived performance among Taekwondo athletes. By highlighting the added value of structured feedback within reflective journaling, rather than the independent effect of journaling alone, this study contributes to the broader sports psychology and self-regulation literature. As interest in supporting athletes’ self-regulation, performance-related self-evaluation, and psychological well-being continues to grow, the findings of this study suggest the potential value of feedback-enhanced reflective journaling in addressing these objectives. Future research should examine the generalizability and long-term effects of feedback-enhanced reflective journaling across diverse sports and populations.

## Data Availability

All data generated or analyzed during this study are included in this published article.

## Abbreviations

ANOVA: Analysis of variance
*t*: *t*-Statistic (from independent *t*-test)
*M*: Mean
*p*: Probability value
*F*: F-statistic (from ANOVA)
χ^2^: Chi-square statistic
SRL-SRS: Self-Regulation of Learning Self-Report Scale
TEOSQ: Task and Ego Orientation in Sport Questionnaire
EFA: Exploratory factor analysis
KMO: Kaiser–Meyer–Olkin
SD: Standard deviation
SS: Sum of squares
MS: Mean square
ηp^2^: partial eta squared

## References

[1] Boud D, Keogh R, Walker D. Reflection: turning experience into learning. London: Kogan Page; 1985.

[2] Berthold K, Nückles M, Renkl A. Do learning protocols support learning strategies and outcomes? The role of cognitive and metacognitive prompts. Learn Instr. 2007;17:564–77. 10.1016/j.learninstruc.2007.09.007.

[3] Nückles M, Roelle J, Glogger-Frey I, Waldeyer J, Renkl A. The self-regulation-view in writing-to-learn: using journal writing to optimize cognitive load in self-regulated learning. Educ Psychol Rev. 2020;32:1089–126. 10.1007/s10648-020-09541-1.

[4] Loughran JJ. Effective reflective practice: in search of meaning in learning about teaching. J Teach Educ. 2002;53:33–43. 10.1177/0022487102053001004.

[5] Ottesen E. Reflection in teacher education. Reflect Pract. 2007;8:31–46. 10.1080/14623940601138899.

[6] Jonker L, Elferink-Gemser MT, de Roos IM, Visscher C. The role of reflection in sport expertise. Sport Psychol. 2012;26:224–42. 10.1123/tsp.26.2.224.

[7] Hutter RI, Oldenhof-Veldman T, Pijpers JR, Oudejans RRD. Professional development in sport psychology: relating learning experiences to learning outcomes. J Appl Sport Psychol. 2017;29:1–16. 10.1080/10413200.2016.1183152.

[8] Anderson AG, Knowles Z, Gilbourne D. Reflective practice for sport psychologists: concepts, models, practical implications, and thoughts on dissemination. Sport Psychol. 2004;18:188–203. 10.1123/tsp.18.2.188.

[9] Elbe AM, Wikman JM. Psychological factors in developing high performance athletes. In: Baker J, Cobley S, Schorer J, Wattie N, editors. Routledge handbook of talent identification and development in sport. London: Routledge; 2017. p. 169–80; 10.4324/9781315668017-12.

[10] Dyment JE, O’Connell TS. Assessing the quality of reflection in student journals: a review of the research. Teach Higher Educ. 2011;16:81–97. 10.1080/13562517.2010.507308.

[11] Wessel J, Larin H. Change in reflections of physiotherapy students over time in clinical placements. Learn Health Soc Care. 2006;5:119–32. 10.1111/j.1473-6861.2006.00124.x.

[12] Jensen SK, Joy C. Exploring a model to evaluate levels of reflection in baccalaureate nursing students’ journals. J Nurs Educ. 2005;44:139–42. 10.3928/01484834-20050301-08. 15787024 10.3928/01484834-20050301-08

[13] Golombek PR. Redrawing the boundaries of language teacher cognition: language teacher educators’ emotion, cognition, and activity. Mod Lang J. 2015;99:470–84. 10.1111/modl.12236.

[14] Kelly EL, Casola AR, Smith K, Kelly S, de la Cruz MSD. A qualitative analysis of third-year medical students’ reflection essays regarding the impact of COVID-19 on their education. BMC Med Educ. 2021;21:481. 10.1186/s12909-021-02906-2. 34496820 10.1186/s12909-021-02906-2 PMC8425993

[15] Țîru CM. Using students’ reflection in the university educational process – a qualitative approach. Educatia. 2021;21:48–61. 10.24193/ed21.2021.21.05.

[16] Richards JC, Farrell TSC. Professional development for language teachers. Cambridge: Cambridge University Press; 2005. 10.1017/CBO9780511667237.

[17] Abrami PC, Wade A, Pillay V, Aslan O, Bures EM, Bentley C. Encouraging self-regulated learning through electronic portfolios. Canadian Journal of Learning and Technology / La revue canadienne de l’apprentissage et de la technologie. 2009;34:1–39. 10.21432/T2630W.

[18] Kicken W, Brand-Gruwel S, van Merriënboer JJG, Slot W. The effects of portfolio-based advice on the development of self-directed learning skills in secondary vocational education. Educ Technol Res Dev. 2009;57:439–60. 10.1007/s11423-009-9111-3.

[19] Chirema KD. The use of reflective journals in the promotion of reflection and learning in post registration nursing students. Doctoral thesis. Huddersfidld: University of Huddersfield; 2003. 10.1016/j.nedt.2006.04.007 16815600

[20] Wong FKY, Kember D, Chung LYF, Yan L. Assessing the level of student reflection from reflective journals. J Adv Nurs. 1995;22:48–57. 10.1046/j.1365-2648.1995.22010048.x. 7560535 10.1046/j.1365-2648.1995.22010048.x

[21] Hattie J, Timperley H. The power of feedback. Rev Educ Res. 2007;77:81–112. 10.3102/003465430298487.

[22] Coulson D, Harvey M. Scaffolding student reflection for experience-based learning: a framework. Teach High Educ. 2013;18:401–13. 10.1080/13562517.2012.752726.

[23] Quinton S, Smallbone T. Feeding forward: using feedback to promote student reflection and learning – a teaching model. Innov Educ Teach Int. 2010;47:125–35. 10.1080/14703290903525911.

[24] Zimmerman BJ. Attaining self-regulation: a social cognitive perspective. In: Boekaerts M, Pintrich PR, Zeidner M, editors. Handbook of self-regulation. California: Academic Press; 2000. p. 13–39.

[25] Inzlicht M, Werner KM, Briskin JL, Roberts BW. Integrating models of self-regulation. Annu Rev Psychol. 2021;72:319–45. 10.1146/annurev-psych-061020-105721. 33017559 10.1146/annurev-psych-061020-105721

[26] Durand-Bush N, Baker J, van den Berg F, Richard V, Bloom GA. The gold medal profile for sport psychology (GMP-SP). J Appl Sport Psychol. 2023;35:547–70. 10.1080/10413200.2022.2055224.

[27] Balk YA, Englert C. Recovery self-regulation in sport: theory, research, and practice. Int J Sports Sci Coach. 2020;15:273–81. 10.1177/1747954119897528.

[28] Ravizza K, Fifer A. Increasing awareness for sport performance. In: Williams JM, Krane V, editors. Applied sport psychology: personal growth to peak performance. 7th ed. New York, NY: McGraw-Hill; 2015.

[29] Dupee M, Forneris T, Werthner P. Perceived outcomes of a biofeedback and neurofeedback training intervention for optimal performance: learning to enhance self-awareness and self-regulation with Olympic athletes. The Sport Psychologist. 2016;30:339–49. 10.1123/tsp.2016-0028.

[30] Beckmann J, Kellmann M. Self-regulation and recovery: approaching an understanding of the process of recovery from stress. Psychol Rep. 2004;95:1135–53. 10.1123/tsp.2016-0028. 15762394 10.2466/pr0.95.3f.1135-1153

[31] Lee ST. Examining the relationships between metacognition, self-regulation and critical thinking in online Socratic seminars for high school social studies students. Ph.D. Dissertation. Austin: The University of Texas at Austin; 2009.

[32] Hashempour M, Ghonsooly B, Ghanizadeh A. A study of translation students’ self-regulation and metacognitive awareness in association with their gender and educational level. Int J Comp Lit Transl Stud. 2015;3:60–9. 10.7575/aiac.ijclts.v.3n.3p.60.

[33] Akcaoğlu MÖ, Mor E, Külekçi E. The mediating role of metacognitive awareness in the relationship between critical thinking and self-regulation. Thinking Skills and Creativity. 2023;47:101187. 10.1016/j.tsc.2022.101187.

[34] Hsu Y, Lin TY, Lu FJH. Beyond the “I” framework: improving emotional expression and increasing social connectedness among college athletes through the psychological displacement paradigm in diary‐writing. Psychol Sch. 2023;60:40–52. 10.1002/pits.22752.

[35] Ullrich PM, Lutgendorf SK. Journaling about stressful events: effects of cognitive processing and emotional expression. Ann Behav Med. 2002;24:244–50. 10.1207/S15324796ABM2403_10. 12173682 10.1207/S15324796ABM2403_10

[36] Lepore SJ, Smyth JM. The writing cure: how expressive writing promotes health and emotional well-being. Washington, DC: American Psychological Association; 2002.

[37] Locke EA, Latham GP. New directions in goal-setting theory. Curr Dir Psychol Sci. 2006;15:265–8. 10.1111/j.1467-8721.2006.00449.x.

[38] Lenzen SA, Daniëls R, van Bokhoven MA, van der Weijden T, Beurskens A. Disentangling self-management goal setting and action planning: a scoping review. PLoS ONE. 2017;12:e0188822. 10.1371/journal.pone.0188822. 29176800 10.1371/journal.pone.0188822 PMC5703565

[39] Popovych I, Semenov O, Hrys A, Aleksieieva M, Pavliuk M, Semenova N. Research on mental states of weightlifters’ self-regulation readiness for competitions. J Phys Educ Sport. 2022;22:1134–44.

[40] Hanrahan SJ, Pedro R, Cerin E. Structured self-reflection as a tool to enhance perceived performance and maintain effort in adult recreational salsa dancers. Sport Psychol. 2009;23:151–69. 10.1123/tsp.23.2.151.

[41] Kim BJ, Gill DL. A cross-cultural extension of goal perspective theory to Korean youth sport. J Sport Exerc Psychol. 1997;19:142–55. 10.1123/jsep.19.2.142.

[42] Balk YA, de Jonge J, Geurts SAE, Oerlemans WGM. Antecedents and consequences of perceived autonomy support in elite sport: a diary study linking coaches’ off-job recovery and athletes’ performance satisfaction. Psychol Sport Exerc. 2019;44:26–34. 10.1016/j.psychsport.2019.04.020.

[43] Passos P, Araújo D, Davids K. Competitiveness and the process of co-adaptation in team sport performance. Front Psychol. 2016;7:1562. 10.3389/fpsyg.2016.01562. 27777565 10.3389/fpsyg.2016.01562 PMC5056172

[44] Beni S, Fletcher T, Ní CD. Meaningful experiences in physical education and youth sport: a review of the literature. Quest. 2017;69:291–312. 10.1080/00336297.2016.1224192.

[45] Thorburn M. Can physical education be meaningful: the role of embodied subjectivity in enhancing self and social learning? Curr Stud Health Phys Educ. 2021;12:53–66. 10.1080/25742981.2020.1844028.

[46] Gennarelli SM, Brown SM, Mulcahey MK. Psychosocial interventions help facilitate recovery following musculoskeletal sports injuries: a systematic review. Phys Sportsmed. 2020;48:370–7. 10.1080/00913847.2020.1744486. 32186423 10.1080/00913847.2020.1744486

[47] Brown DJ, Fletcher D. Effects of psychological and psychosocial interventions on sport performance: a meta-analysis. Sports Med. 2017;47:77–99. 10.1007/s40279-016-0552-7. 27241124 10.1007/s40279-016-0552-7

[48] Lochbaum M, Zanatta T, Kirschling D, May E. The profile of moods states and athletic performance: a meta-analysis of published studies. Eur J Investig Health Psychol Educ. 2021;11:50–70. 10.3390/ejihpe11010005. 34542449 10.3390/ejihpe11010005 PMC8314345

[49] Swann C, Crust L, Jackman P, Vella SA, Allen MS, Keegan R. Psychological states underlying excellent performance in sport: toward an integrated model of flow and clutch states. J Appl Sport Psychol. 2017;29:375–401. 10.1080/10413200.2016.1272650.

[50] Anderson R, Hanrahan SJ, Mallett CJ. Investigating the optimal psychological state for peak performance in Australian elite athletes. J Appl Sport Psychol. 2014;26:318–33. 10.1080/10413200.2014.885915.

[51] Schinke RJ, Giffin C, Cosh S, Douglas K, Rhind D, Harwood C, et al. International society of sport psychology position stand: mental health through occupational health and safety in high performance sport. Int J Sport Exerc Psychol. 2022;20:1711–33. 10.1080/1612197X.2021.1992857.

[52] González-Hernández J, Gomariz-Gea M, Valero-Valenzuela A, Gómez-López M. Resilient resources in youth athletes and their relationship with anxiety in different team sports. Int J Environ Res Public Health. 2020;17:5569. 10.3390/ijerph17155569. 32752247 10.3390/ijerph17155569 PMC7432103

[53] Doorley JD, Kashdan TB. Positive and negative emotion regulation in college athletes: a preliminary exploration of daily savoring, acceptance, and cognitive reappraisal. Cognit Ther Res. 2021;45:598–613. 10.1007/s10608-020-10202-4. 33518842 10.1007/s10608-020-10202-4 PMC7821841

[54] Sun S, Zhang SX, Jahanshahi AA, Jahanshahi M. Drilling under the COVID‐19 pandemic: a diary study of professional football players’ mental health and workout performance. Stress Health. 2022;38:3–18. 10.1002/smi.3059. 33945206 10.1002/smi.3059 PMC8237014

[55] Seok R. Analysis of reflective thinking, self-direction and psychological technique effectiveness through reflective training journal writing of Taekwondo athletes. Doctoral dissertation. Kyung Hee University; 2016. https://khu.dcollection.net/public_resource/pdf/200000056502_20241228151312.pdf.

[56] Gibbs G. Learning by doing: a guide to teaching and learning methods. Oxford: Further Education Unit. Oxford Polytechnic; 1988.

[57] Toering T, Elferink-Gemser MT, Jonker L, van Heuvelen MJG, Visscher C. Measuring self-regulation in a learning context: reliability and validity of the Self-Regulation of Learning Self-Report Scale (SRL-SRS). Int J Sport Exerc Psychol. 2012;10:24–38. 10.1080/1612197X.2012.645132.

[58] Kim H-J, Kang SW, Kwon SH. Verification of the structural relationship among athlete Julsil, self-regulation, and flow in adolescent athletes. Int J Appl Sports Sci. 2019;31:13–24. 10.24985/ijass.2019.31.1.13.

[59] Duda JL, Nicholls JG. Dimensions of achievement motivation in schoolwork and sport. J Educ Psychol. 1992;84:290–9. 10.1037/0022-0663.84.3.290.

[60] Hersey P, Blanchard KH. Management of organizational behavior. Englewood Cliffs, NJ: Prentice Hall; 1977.

[61] Nunnally JC, Bernstein IH. Psychometric theory. New York, NY: McGraw-Hill; 1994.

[62] Field A. Discovering statistics using IBM SPSS statistics. London: Sage Publications; 2013.

[63] Asparouhov T, Muthén B. Exploratory structural equation modeling. Struct Equ Modeling. 2009;16:397–438. 10.1080/10705510903008204.

[64] Gorsuch RL. Factor analysis. Hillsdale, NJ: Erlbaum; 1983.

[65] Kaiser HF. An index of factorial simplicity. Psychometrika. 1974;39:31–6. 10.1007/BF02291575.

[66] Van Prooijen JW, van der Kloot WA. Confirmatory analysis of exploratively obtained factor structures. Educ Psychol Meas. 2001;61:777–92. 10.1177/00131640121971518.

[67] Hair JF, Black WC, Babin BJ, Anderson RE. Multivariate data analysis. Upper Saddle River, NJ: Pearson; 2010.

[68] Yap BW, Sim CH. Comparisons of various types of normality tests. J Stat Comput Simul. 2011;81:2141–55. 10.1080/00949655.2010.520163.

[69] Öztuna D, Elhan A, Tüccar E. Investigation of four different normality tests in terms of type 1 error rate and power under different distributions. Turk J Med Sci. 2006;36:171–6.

[70] Farrand P, Perry J, Linsley S. Enhancing self-practice/self-reflection (SP/SR) approach to cognitive behaviour training through the use of reflective blogs. Behav Cogn Psychother. 2010;38:473–7. 10.1017/S1352465810000238. 20459878 10.1017/S1352465810000238

[71] Germain A, Nolan K, Doyle R, Mason S, Gambles M, Chen H, et al. The use of reflective diaries in end of life training programmes: a study exploring the impact of self-reflection on the participants in a volunteer training programme. BMC Palliat Care. 2016;15:28. 10.1186/s12904-016-0096-5. 26944056 10.1186/s12904-016-0096-5 PMC4779245

[72] Kreibich A, Wolf BM, Bettschart M, Ghassemi M, Herrmann M, Brandstätter V. How self-awareness is connected to less experience of action crises in personal goal pursuit. Motiv Emot. 2022;46:825–36. 10.1007/s11031-022-09942-5. 36439374 10.1007/s11031-022-09942-5 PMC9678988

[73] Young BW, Wilson SG, Hoar S, Bain L, Siekańska M, Baker J. On the self-regulation of sport practice: moving the narrative from theory and assessment toward practice. Front Psychol. 2023;14:1089110. 10.3389/fpsyg.2023.1089110. 37057149 10.3389/fpsyg.2023.1089110 PMC10086193

[74] Baumeister RF, Vohs KD, Tice DM. The strength model of self-control. Curr Dir Psychol Sci. 2007;16:351–5. 10.1111/j.1467-8721.2007.00534.x.

[75] Duckworth AL, Gendler TS, Gross JJ. Situational strategies for self-control. Perspect Psychol Sci. 2016;11:35–55. 10.1177/1745691615623247. 26817725 10.1177/1745691615623247 PMC4736542

[76] Berkman ET, Hutcherson CA, Livingston JL, Kahn LE, Inzlicht M. Self-control as value-based choice. Curr Dir Psychol Sci. 2017;26:422–8. 10.1177/0963721417704394. 29335665 10.1177/0963721417704394 PMC5765996

[77] Gillebaart M. The ‘operational’ definition of self-control. Front Psychol. 2018;9:1231. 10.3389/fpsyg.2018.01231. 30072939 10.3389/fpsyg.2018.01231 PMC6058080

[78] Brydges R, Butler DL. A reflective analysis of medical education research on self‐regulation in learning and practice. Med Educ. 2012;46:71–9. 10.1111/j.1365-2923.2011.04100.x. 22150198 10.1111/j.1365-2923.2011.04100.x

[79] Kehr HM, Bles P, Rosenstiel L. Self-regulation, self-control, and management training transfer. Int J Educ Res. 1999;31:487–98. 10.1016/S0883-0355(99)00017-8.

[80] Button C, Seifert L, Chow JY, Araújo D, Davids K. Dynamics of skill acquisition: an ecological dynamics approach. Champaign, IL: Human Kinetics Publishers; 2021.

[81] Seufert T. Building bridges between self-regulation and cognitive load—an invitation for a broad and differentiated attempt. Educ Psychol Rev. 2020;32:1151–62. 10.1007/s10648-020-09574-6.

[82] Balk YA, de Jonge J, Oerlemans WGM, Geurts SAE. Testing the triple-match principle among Dutch elite athletes: a day-level study on sport demands, detachment and recovery. Psychol Sport Exerc. 2017;33:7–17. 10.1016/j.psychsport.2017.07.006.

[83] Kellmann M. Preventing overtraining in athletes in high‐intensity sports and stress/recovery monitoring. Scand J Med Sci Sports. 2010;20(2):95–102. 10.1111/j.1600-0838.2010.01192.x. 20840567 10.1111/j.1600-0838.2010.01192.x

[84] Almagro BJ, Sáenz-López P, Fierro-Suero S, Conde C. Perceived performance, intrinsic motivation and adherence in athletes. Int J Environ Res Public Health. 2020;17:9441. 10.3390/ijerph17249441. 33339278 10.3390/ijerph17249441 PMC7767293

